# The circular RNA circZFR phosphorylates Rb promoting cervical cancer progression by regulating the SSBP1/CDK2/cyclin E1 complex

**DOI:** 10.1186/s13046-021-01849-2

**Published:** 2021-01-30

**Authors:** Mingyi Zhou, Zhuo Yang, Danbo Wang, Peng Chen, Yong Zhang

**Affiliations:** 1 Department of Gynecology, Cancer Hospital of China Medical University, Liaoning Cancer Hospital & Institute, Shenyang, 110042 Liaoning Province People’s Republic of China; 2 Department of Pathology, Cancer Hospital of China Medical University, Liaoning Cancer Hospital & Institute, Shenyang, 110042 China

**Keywords:** Circular RNAs, Cervical cancer, Cell cycle regulation, RNA Immunoprecipitation, RNA binding protein

## Abstract

**Background:**

As a novel type of non-coding RNA, circular RNAs (circRNAs) play a critical role in the initiation and development of various diseases, including cancer. However, the exact function of circRNAs in human cervical cancer remains largely unknown.

**Methods:**

We identified the circRNA signature of upregulated circRNAs between cervical cancer and paired adjacent normal tissues. Using two different cohorts and GEO database, a total of six upregulated circRNAs were identified with a fold change > 2, and *P* < 0.05. Among these six circRNAs, hsa_circ_0072088 (circZFR) was the only exonic circRNA significantly overexpressed in cervical cancer. Functional experiments were performed to investigate the biological function of circZFR. **Circ**RNA pull-down, circRNA immunoprecipitation (circRIP) and Co-immunoprecipitation (Co-IP) assays were executed to investigate the molecular mechanism underlying the function of circZFR.

**Results:**

Functionally, circZFR knockdown represses the proliferation, invasion, and tumor growth. Furthermore, circRNA pull-down experiments combined with mass spectrometry unveil the interactions of circZFR with Single-Stranded DNA Binding Protein 1 (SSBP1). Mechanistically, circZFR bound with SSBP1, thereby promoting the assembly of CDK2/cyclin E1 complexes. The activation of CDK2/cyclin E1 complexes induced p-Rb phosphorylation, thus releasing activated E2F1 leading to cell cycle progression and cell proliferation.

**Conclusion:**

Our findings provide the first evidence that circZFR is a novel onco-circRNA and might be a potential biomarker and therapeutic target for cervical cancer patients.

**Supplementary Information:**

The online version contains supplementary material available at 10.1186/s13046-021-01849-2.

## Background

Despite the human papilloma virus (HPV) vaccine and screening, cervical cancer remains one of the fourth most frequent cancer in women worldwide [1, 2]. The morbidity and mortality of cervical cancer in low-income and lower-middle-income countries remain high [3]. Moreover, an emerging number of young women have cervical cancer and impair their fertility [4]. Despite surgical therapy, chemotherapy, and radiotherapy, cervical cancer still exhibits high invasion and mortality rates.

Recently, non-coding RNAs (ncRNA) has become a hot-spot as a critical regulator in tumorigenesis [5]. Circular RNAs (circRNAs), are a new class of endogenous functional ncRNAs and derived from precursor mRNA back splicing to form a continuous closed loop structure, are more stable and resistant to degradation than liner RNAs [6–8]. Emerging evidence has suggested that circRNAs can participate in the development and progression of malignant tumors [6]. However, the exact mechanism is still poorly understood. One molecular mechanism involves cytoplasmic circRNAs acting as competitive endogenous RNA (ceRNA), such as microRNAs (miRNAs) sponge that represses protein-coding mRNAs by binding to miRNA response elements (MREs) and thus, can reduce their capacity to bind to and downregulate protein transcripts [9]. Others circular RNAs can interact with proteins as RNA-binding proteins (RBPs), via specific RNA-binding domains (RBDs), to form RNA-protein complex and regulate their activities in human cancers. Currently, more than 1500 RBPs and several kinds of RBDs have been identified [10–13].

The complexity of the circRNAs regulatory functions remains to be determined, particularly during the cell cycle and the proliferation of cervical cancer. Moreover, circRNAs have high tissue specificity. Because of their stability, resistance to degradation, and tissue specificity, circRNAs may represent novel biomarkers or therapies for specific cancer [9, 14–16].

Here, we aim to investigate the function of circRNAs in regulating the proliferation of cervical cancer. We identified a circRNAs signature of six upregulated circRNAs between cervical cancer tissues and paired non-cancer tissues, validated our results from the GEO database, and found that hsa_circ_0072088 (circZFR) was the only exonic circRNA significantly upregulated in squamous cervical cancer. Mechanistically, Single-Stranded DNA Binding Protein 1 (SSBP1), which is a candidate RBP, was identified by RNA pull-down and circRNA-immunoprecipitation assay (circRIP) to interact with circZFR. The SSBP1-circZFR complex recruits and activates the CDK2/cyclin-E1 complex driving Rb phosphorylation leading the inactivation of retinoblastoma protein and therefore releasing E2F1 transcription factor. Thus, leading to E2F1 transcription factor to express genes that promote entry into S-phase and promotes proliferation of cervical cancer. In conclusion, our results suggested that circZFR is a novel onco-circRNA and a potential biomarker for cervical cancer.

## Methods

### Cervical tumors samples and cell cultures

In this study, 30 pairs of cervical cancer and adjacent normal tissues, ten advanced cervical cancer without adjacent normal tissue, and seven normal cervical epithelial tissues of the patients who had a hysterectomy due to myoma were obtained from Liaoning Cancer Hospital. Any cases that received chemotherapy or radiotherapy before collection were excluded. Only squamous cervical cancer cases were collected in order to decrease heterogeneity due to different histological types.

Human cervical cancer cell lines (HeLa and SiHa) were purchased from the Shanghai Institutes for Biological Sciences, China. All cell lines were cultured in Dulbecco’s Modified Eagle’s medium (DMEM) high glucose medium (HyClone, Logan, UT, United States) supplemented with 10% fetal bovine serum (FBS).

### circRNAs expression profile analysis

We performed circRNA microarray analysis (human CircRNA microarray V2.0) using three cervical cancer tissues and paired adjacent normal tissues in our Liaoning Cancer Hospital & Institute (LCHI)‘s cohort. Another profile using the same microarray (GSE102686) was downloaded from the Gene Expression Omnibus database (GEO, http://www.ncbi.nlm.nih.gov/geo). GSE102686 consisted of 5 cervical cancer tissues and paired adjacent normal tissues. R version 3.6.2 software (https://www.r-project.org/) was used to compare the differentially expressed genes (DEGs) of two profiles separately.

### TCGA and GTEx data analyses

A total of 306 cervical cancer and 13 healthy cervical tissues and the corresponding clinical data were obtained from The Cancer Genome Atlas (TCGA, https://cancergenome.nih.gov/) and Genotype-Tissue Expression (GTEx, https://www.gtexportal.org/home/index.html). All the data included in this study are in agreement with the TCGA and GTEx publication guidelines.

### Quantitative reverse transcription-polymerase reaction (qRT-PCR)

Total RNA from tissues and cells was isolated using TRIZOL reagent (Invitrogen, CA, USA). For circRNA and mRNA, cDNA was synthesized using the PrimeScript RT reagent Kit with gDNA Eraser (Takara, Otsu, Japan). The quantification of circRNA and mRNA was performed using SYBR Premix Ex Taq II (Takara, Otsu, Japan). CircZFR and mRNA expression were detected using the specific primer pairs (Supplementary Table 1). β-actin was used as the internal reference for the quantification of circRNA and mRNA. qRT-PCR was conducted on the Bio-Rad CFX96 system (Bio-Rad, CA, USA). The relative expression of circRNAs and mRNAs was calculated with the 2-ΔΔCT method.

### CircRNA plasmid construction and stable cell lines

Stable cell lines expressing CircZFR or sh-CircZFR and controls (CircCtrl and sh-Ctrl) were performed as previously described [17]. In brief, recombinant lentiviruses were produced in HEK293 cells. The viruses were harvested and purified by centrifugation. Pools of stable transductions were generated by selection using puromycin (1.0 μg/ml HeLa and 5.0 μg/ml for SiHa) for two weeks**.** The sequences of siRNAs and shRNAs targeting circZFR are listed in Supplementary Table 2.

### SSBP1 plasmid construction

The target sequences of SSBP1 shRNAs are listed in Supplementary Table 3. The shRNA with the most significant knockdown efficiency was selected in this study.

### E2F1 siRNAs

The siRNAs for E2F1 were synthesized from Ambion (Austin, TX, USA). The sequences of E2F1 siRNAs are listed in Supplementary Table 4.

### Cell proliferation and cell cycle analyses

We performed cell counting and 5-Ethynyl-20-deoxyuridine (EdU) assays to assess cell proliferation. The cell counting assay was performed using Cell Counting Kit-8 (Dojindo Laboratories, Kumamoto, Japan) as previously described [17]. The stably transfected cells (2.0 × 10^3^/well) were plated into 96-well plates and incubated at 37 °C for 8 h until adherence and were referred to as day 0. Cell numbers were counted on days 1, 2, 3, and 4. The EdU incorporation assay was performed using a Cell-Light EdU DNA Cell Proliferation Kit (RiboBio, Guangzhou, China) according to the manufacturer’s protocol [18]. The percentage of EdU-positive cells was calculated. We also performed a colony formation assay to validate the function of circZFR on cervical cancer cell proliferation. Total of 500 stably transfected cells were seeded into six-well plates. The plates were photographed and counted after 14 days.

Cell cycle analyses were performed as previously described [19]. Briefly, all of the cells were first synchronized through serum deprivation for 36 h. The cells were stained using PI/RNase Staining Buffer (BD BioScience) and then analyzed in BD Accuri™ C6 (BD BioScience).

### Wound healing, migration, and invasion assays

Wound healing, migration, and invasion assays were performed as previously described [20]. The stably transfected cells were cultured in 6-well plates, for wound healing assay, until 90–100% confluent. After scratching, the cells were washed and maintained in serum-free medium. At 0, 12, and 24 h three-time points after wounding, the width of wounds was examined in three-independent wound sites per group and normalized to a control group.

Migration and invasion assays were performed using transwell chambers, which contain inserts with a pore size of 8 μm (Corning Incorporated, Corning, NY, USA). Total 2 × 10^3^ stably transfected cells were plated into an upper chamber coated with or without Matrigel (Corning Incorporated, Corning, NY, USA) with serum-free medium, and the lower chamber was added 10% fetal bovine serum. After incubation for 24 h, the cells passed through the membrane were fixed, stained, and counted in 10 randomly selected fields with a 200x magnification microscope (Leica DMi8, Wetzlar, Germany).

### Western blot and immunohistochemistry

Western Blot analyses were performed according to the published protocol [21]. Cells and tumor tissues were lysed by RIPA lysis buffer with protease and phosphates inhibitor. Then the lysates were resolved by 8–12% SDS-PAGE and transferred on polyvinylidene fluoride membranes (Millipore, Bedford, MA, USA). After blocking, the membranes were immunoblotted with primary antibodies against p-Rb S807 (ab184796, 1:1000, Abcam, Cambridge, UK), p-Rb S608 (ab172975, 1:20000), p-Rb S780 (ab173289, 1:10000), p-Rb T821 (ab32015, 1:5000), p-Rb (ab181616, 1:2000), E2F1 (ab179445, 1:2000), cyclin E1 (ab33911, 1:2000), CDK4 (ab108357, 1:5000), CDK2 (ab32147, 1:5000), ac-E2F1 K117 (YK0087, 1:2000, Immunoway, Texas, USA), ac-E2F1 K125 (YK0088, 1:1000), cyclin D1 (60186–1-Ig, 1:10000, Proteintech, Wuhan, China), SSBP1 (12212–1-AP, 1:1000, Proteintech), and β–actin (60008–1-Ig, 1:20000, Proteintech) overnight at 4 °C. After the incubation with secondary antibodies, the signals were developed with chemiluminescent western blotting substrate (Beyotime, Shanghai, China). ImageJ software was used to quantify signal intensity.

For immunohistochemical staining, total 30 pairs of formalin-fixed, paraffin-embedded cervical cancer and adjacent normal tissue specimens were analyzed. Briefly, after dewaxing the paraffin sections of tissues, heat-induced antigen retrieval was conducted. The slides were incubated with primary antibodies against p-Rb S807 (ab184796, 1:200) and p-Rb S608 (ab172975, 1:50) at 4 °C overnight, incubated with the biotinylated secondary antibody, and then subjected to the DAB kit. The evaluation of p-Rb S807 and S608 phosphorylation protein expression levels was performed as described previously [20].

### circRNA pull-down and circRNA immunoprecipitation (circRIP) assays

MS2 bacteriophage coat protein (MS2-CP) circRNA pull-down assay was performed using the MS2 tagging technique, which is based on the natural binding between a stem-loop structure of MS2 and MS2-CP [22]. In brief, we constructed the plasmid with circZFR and MS2, which was fused with a green fluorescent protein (GFP) (circZFR-MS2^GFP^). We also constructed the plasmid with MS2-CP-Flag, which was fused with mCherry tag (MS2-CP-Flag^mCherry^). HeLa cells were transfected with these two plasmids and precipitated circZFR through pulling down using anti-Flag antibodies. As controls, the lysates derived from the cells without the MS2 tagging system were used. The cell lysates were incubated with Protein A/G beads overnight at 4 °C. After washing, circZFR-MS2-bound proteins were eluted with urea buffer supplemented with dithiothreitol, and trypsin and LysC as previously described [23]. Next, the RNA and bound proteins were eluted with the HiPure Total RNA Mini Kit (MAGEN, Guangzhou, China). RNA was reverse transcribed and analyzed by qPCR, as described above. The bound proteins were analyzed by label-free mass spectrometry (MS).

The circRIP assay was performed with BersinBioTM RNA Immunoprecipitation Kit (BersinBio, Guangzhou, China) according to manufacturer instructions. HeLa and SiHa cells stably overexpressing circ-ZFR or circ-control were used. Briefly, cells were lysed using the complete RNA lysis buffer and then incubated with the RIP buffer containing the magnetic beads conjugated with SSBP1 antibodies (12212–1-AP, 1:1000, Proteintech) or negative control IgG at 4 °C, overnight; then, the beads were washed three times. After Proteinase K treatment, the immunoprecipitated RNAs were extracted using phenol-chloroform-isoamylol (25:24:1). Finally, qRT-PCR was performed to identify the expression of circZFR.

### Co-immunoprecipitation (co-IP)

The Co-IP assay involving CDK2 and SSBP1 was performed using Pierce Crosslink Immunoprecipitation Kit (Pierce, Rockford, IL) according to the manufacturer’s instructions. Briefly, cells were serum-starved for 36 h prior to cell lysis. Then antibodies cross-linked Protein A/G Plus-Agarose were added, and the eluted samples were analyzed by western blot, as described above.

### Animal models

Stably-transfected HeLa cells (2 × 10^7^ cells) were subcutaneously injected into 4 to 6-week-old female BALB/c nude mice (Beijing HFK Bioscience, Beijing, China). The length and width of the subcutaneous tumor were measured once 3 three days, and the volume of the subcutaneous tumor was calculated according to this formula: (length×width^2^)/2. After 21 days, the mice were sacrificed, imaged, and the weight of tumors was measured. The animal procedures were approved by the Institutional Animal Care and Use Committees of China Medical University.

### Statistical analysis

Data were presented as the mean ± standard deviation (SD) from three independent experiments. A paired t-test (two-tailed) was used to analyze the differences in circZFR levels between cervical cancer and paired normal cervical tissues. Other differences between the two groups were analyzed using the Student’s t-test (two-tailed) or Chi-square test. Pearson’s correlation coefficient analysis was used to analyze the correlations. *P* < 0.05 was considered statistically significant. The statistical analyses were conducted with SPSS19.0 (Chicago, IL, USA) or GraphPad Prism 7.0 (La Jolla, CA, USA).

## Results

### CircZFR was significantly upregulated in cervical cancer

**Fig. 1:**
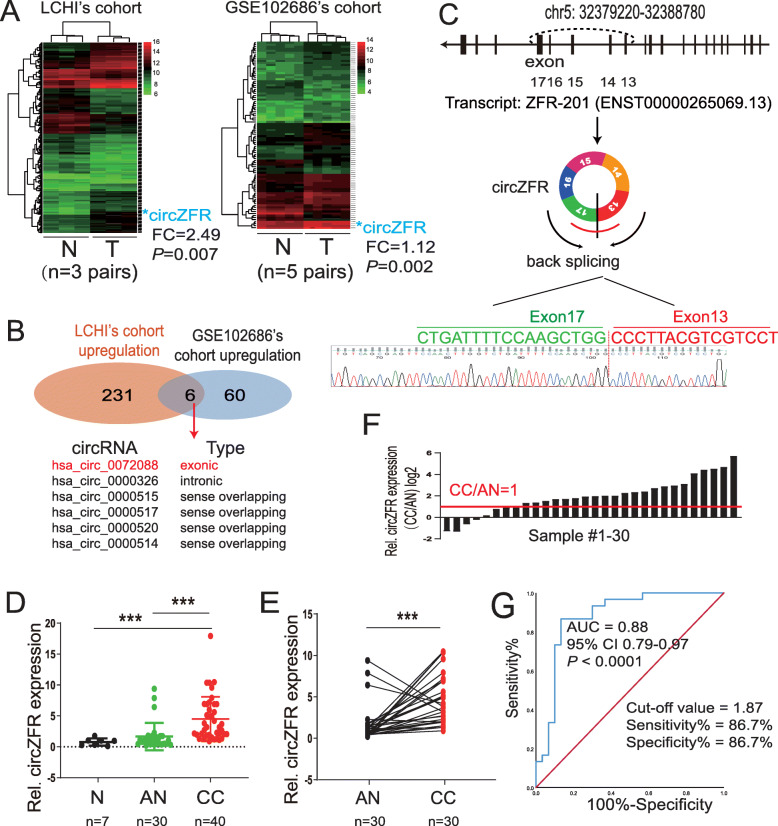
Upregulation of CircZFR in cervical cancer tissues. **a.** CircRNAs expression profiling. Heatmap of upregulated circRNAs profiles in cervical cancer tissues (T) vs. paired adjacent non-cancer tissues (N), according to our LCHI’s cohort and GSE102686 data from the GEO database. CircZFR (hsa_circ_0072088, hsa_circRNA_103809) is significantly upregulated for cervical cancer in both cohorts. LCHI: Liaoning Cancer Hospital & Institute, FC: fold change **b.** Venn diagram. **c.** The genomic loci of *ZFR* gene and Sanger sequencing for the PCR products using divergent circZFR primers confirmed the head-to-tail splicing (Exon 13 and 17). **d.** The level of circZFR was significantly higher in cervical cancer (CC) than adjacent normal (AN) and normal cervix (N), as determined by qRT-PCR. **e.** The differential expression of circZFR in most cervical cancer (CC) was higher than adjacent normal tissues (AN) (86.7%, 26/30). **f.** qRT-PCR for the abundance of circZFR in 30 patients with cervical cancer and adjacent normal. **g.** The diagnostic value of circZFR for cervical cancer was evaluated by receiver operating characteristic (ROC) analysis. ***P< 0.001. AUC: area under the curve, CI: confidence interval

To determine the circRNAs expression profiling in the progression of cervical cancer, we performed circRNA microarray analysis (human CircRNA microarray V2.0) using three cervical cancer tissues and paired adjacent normal tissues [24]. A total of 237 circRNAs were identified as upregulated in cervical cancer compared to adjacent normal tissues in our LCHI’s cohort (> 2-fold change, *P* < 0.05) **(**Fig. 1a). To validate our results, we also analyzed the differential expression of circRNAs from the GEO database and found the GSE102686 cohort, which compared the circRNAs expression between five pairs of cervical cancer tissues vs. paired adjacent non-cancerous tissues [25] **(**Fig. 1a)**.** Furthermore, a total of 66 upregulated circRNAs were identified in cervical cancer in the GSE 102686’s cohort. Finally, six circRNAs were found to be overexpressed in both cohorts with fold change > 2 between cancer tissues and normal tissues with lower *P* < 0.01 **(**Fig. 1 and S1a)**.** Among these six circRNAs, the only circZFR was identified as an exonic type **(**Fig. 1b). CircZFR [also called hsa_circ_0072088 or hsa_circZFR_032, according to the annotation of circBase (http://www.circbase.org/) and circBank (http://www.circbank.cn/index.html)], was spliced from the *ZFR* gene located at chr5: 32379220–32,388,780 and formed a circular transcript of 693 nt. The divergent primers spanning the circZFR junction amplified the PCR products, and the head-to-tail splicing was confirmed through Sanger sequencing **(**Fig. 1c and S1b). Using circZFR-specific primers, we tested the expression of circZFR in 30 early-stage cervical cancer tissues and paired normal tissues, ten advanced-stage cervical cancer, and seven normal cervical epithelial tissues derived from the patients who had a hysterectomy due to myoma. The qRT-PCR results showed that circZFR expression in cervical cancer was higher than normal cervical tissues (86.7%, 26/30) (*P* < 0.001) **(**Fig. 1d-f**)**. Clinicopathological features of 40 cervical cancer patients (30 with and ten without paired normal tissues) showed that increased expression of circZFR was positively associated with lymphatic metastasis (Table 1, *P* =0.049), squamous cell carcinoma antigen (SCC Ag) value (Table 1, *P* =0.049), and Ki67 value (Table 1, *P* =0.003). However, circZFR was not associated with age, tumor stage, invasion depth, or vascular invasion (Table 1**)**. Furthermore, the area under the receiver operating characteristic (ROC) curve (AUC) for the differentiating value of circZFR in cervical cancer patients from normal was calculated to be 0.88, and the cut-off value was 1.87 with a sensitivity of 86.7% and specificity of 86.7%, suggesting a good predictive value of circZFR expression in diagnosis of cervical cancer (Fig. 1g).

**Table 1:** Relationship between the circZFR expression and clinical parameters of cervical cancer patients

Variable		Case No	Mean ± SD	***P*** value
Age (years)	≤50	20	4.14±2.75	0.535
	> 50	20	4.86±4.34	
Tumor stage	≤IB2+IIA1	32	3.50±2.37	0.389
	>IB2	8	4.75±3.84	
Grade	G2	16	4.24±4.11	0.463
	G3	13	5.33±3.68	
Invasion depth	< 2/3	27	3.91±2.98	0.481
	≥2/3	13	4.78±3.89	
Lymphatic metastasis	Negative	33	4.34±2.94	0.049
	Positive	4	8.11±7.02	
Vascular Invasion	Negative	30	4.70±3.72	0.856
	Positive	7	4.98±3.49	
SCC Ag (ng/mL)	< 1.5	19	3.44±2.53	0.049
	≥1.5	21	5.67±4.28	
Ki67	< 80%	14	2.89±2.48	0.003
	≥80%	13	6.22±2.83	

*G2* grade 2, *G3* grade 3, *SCC Ag* squamous cell carcinoma antigen

### CircZFR promoted cell proliferation, migration, and invasion of cervical cancer cells

**Fig. 2:**
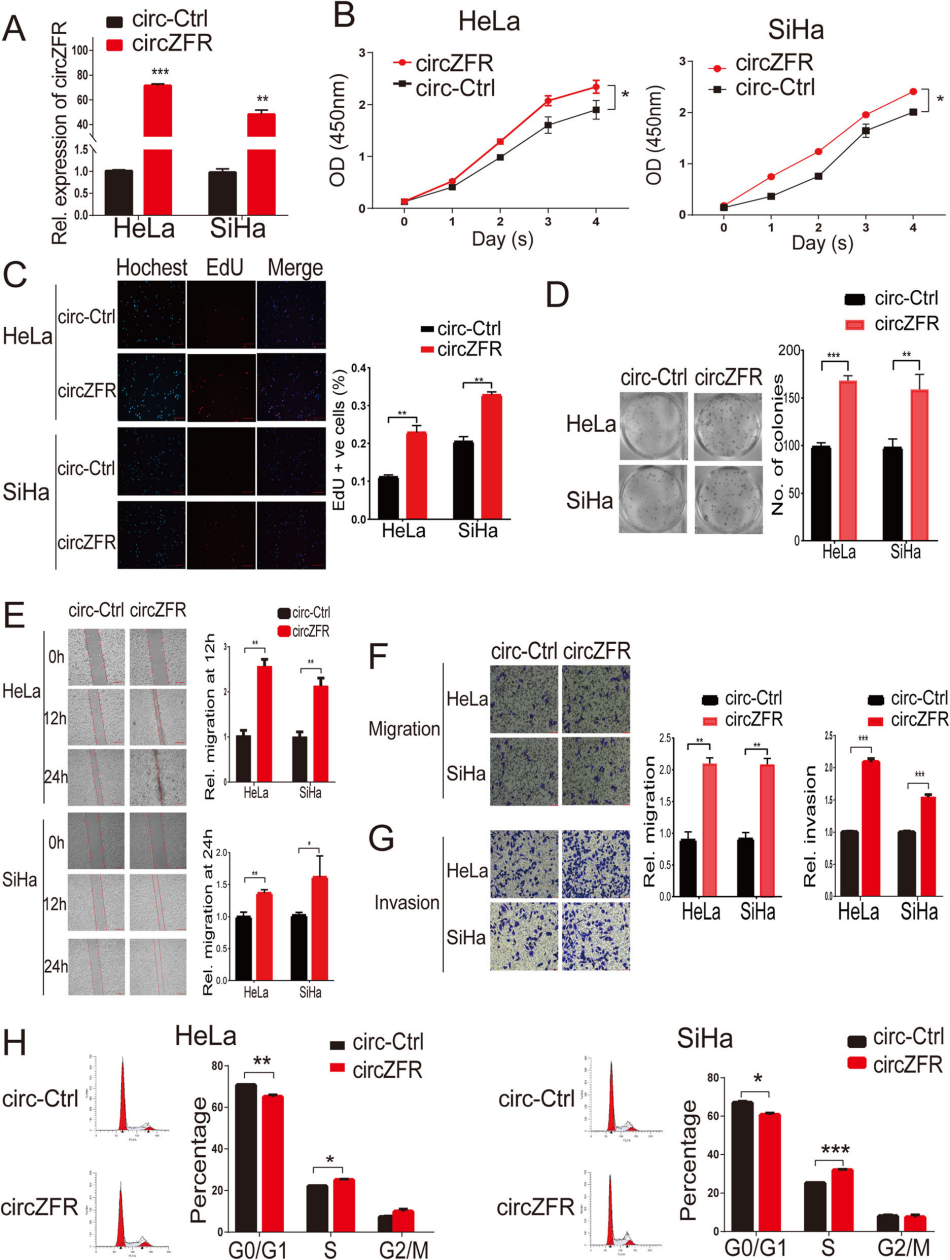
CircZFR promoted proliferation, migration, and invasion of cervical cancer cells. **a.** qRT-PCR analysis of circZFR expression in HeLa and SiHa cells stably expressing circZFR or control-circZFR (circ-Ctrl). **b-c.** Upregulation of circZFR by stable expression of circZFR increases the proliferation ability of HeLa and SiHa cells as measured by CCK-8 assay and EdU assays (Scale bar = 200 μm). **d.** The colony formation assay shows that stable overexpression of circZFR increased the clonogenic ability of HeLa and SiHa cells. **e-g.** The wound healing and transwell matrigel migration and invasion assays show that stable overexpression of circZFR enhanced cell migration and invasion of HeLa and SiHa cells (Scale bar in **e** = 200 μm, scale bar in **f** and **g** = 50 μm). **h.** Significant decrease in the number of cells in the G0-G1 phase of the cell cycle and increased cells in the S phase after overexpression of circZFR as detected by Flow Cytometry (FCM). *P< 0.05, **P< 0.01, ***P< 0.001

**Fig. 3:**
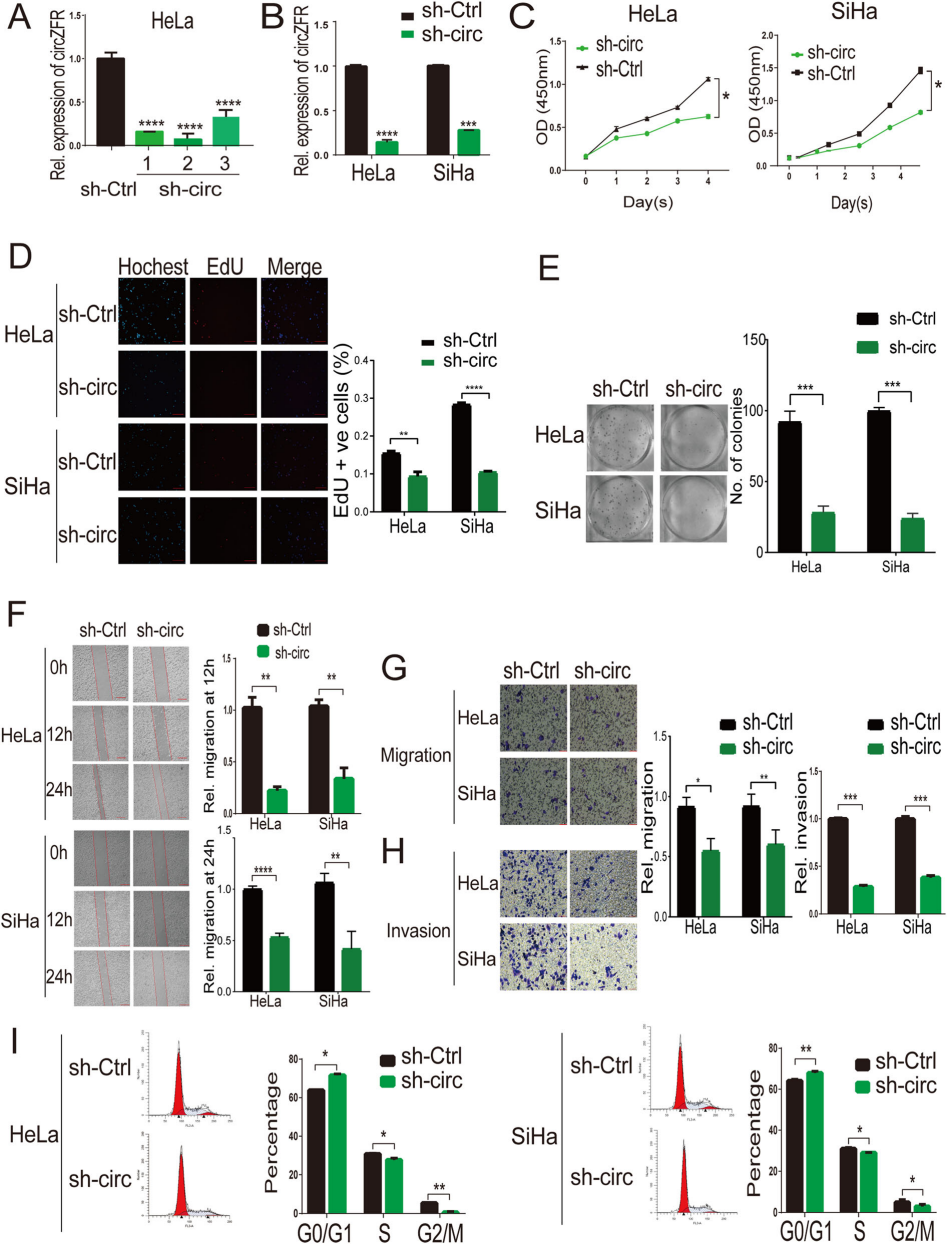
Downregulation of circZFR inhibits proliferation, migration, and invasion of cervical cancer cells. **a.** qRT-PCR analysis of circZFR expression in HeLa cells after stable cirZFR knockdown using three different shRNAs targeting the junction region of circZFR (sh-circ) or control-shRNA (sh-Ctrl). Sh2-circ (sh-circ, hereafter) shown the highest knockdown efficacy. **b.** CircZFR expression levels in HeLa and SiHa cells stably expressing sh-circ or sh-Ctrl. **c-d.** Downregulation of circZFR by stable expression of sh-circZFR inhibits the proliferation ability of HeLa and SiHa cells as measured by CCK-8 assay and EdU assays (Scale bar = 200 μm). **e.** The colony formation assay showed that stable knockdown of circZFR inhibits the clonogenic ability of HeLa and SiHa cells. **f-h.** Stable knockdown of circZFR impedes cell migration and invasion ability of HeLa and SiHa cells as measured by wound healing and transwell matrigel migration and invasion assays (Scale bar in **f** = 200 μm, scale bar in **g** and **h** = 50 μm). **i.** The cell cycle was analyzed using flow cytometry after stable circZFR knockdown. CircZFR knockdown arrested cells in G0-G1 phase and decrease S phase. *P < 0.05, **P< 0.01, ***P< 0.001, ****P< 0.0001

To investigate the biological effect of circZFR on cervical cancer cells, we established a stable expression of circZFR in HeLa and SiHa cells (Fig. 2a). CCK-8 assay, EdU assay, colony formation assay, wound healing and transwell matrigel migration and invasion assays showed that stably circZFR overexpressing cells (HeLa and SiHa cells) were significantly more likely to exhibit a malignant phenotype than control cells (Fig. 2b-g). The cell cycle analysis revealed a significantly increased number of cells in the S phase and decreased in the number of cells in G0-G1 phase significantly after overexpression of circZFR (Fig. 2h). Conversely, reduced circZFR expression by shRNA inhibited cell proliferation, migration, and invasion in HeLa and SiHa cells (Fig. 3a-h). Analysis of the cell cycle assay revealed a significantly increased number of cells in the G0-G1 phase and a significantly decreased number in the S phase after inhibition of circZFR (Fig. 3i).

### CircZFR activating Rb-E2F1 pathway promoted cervical cancer progression

**Fig. 4:**
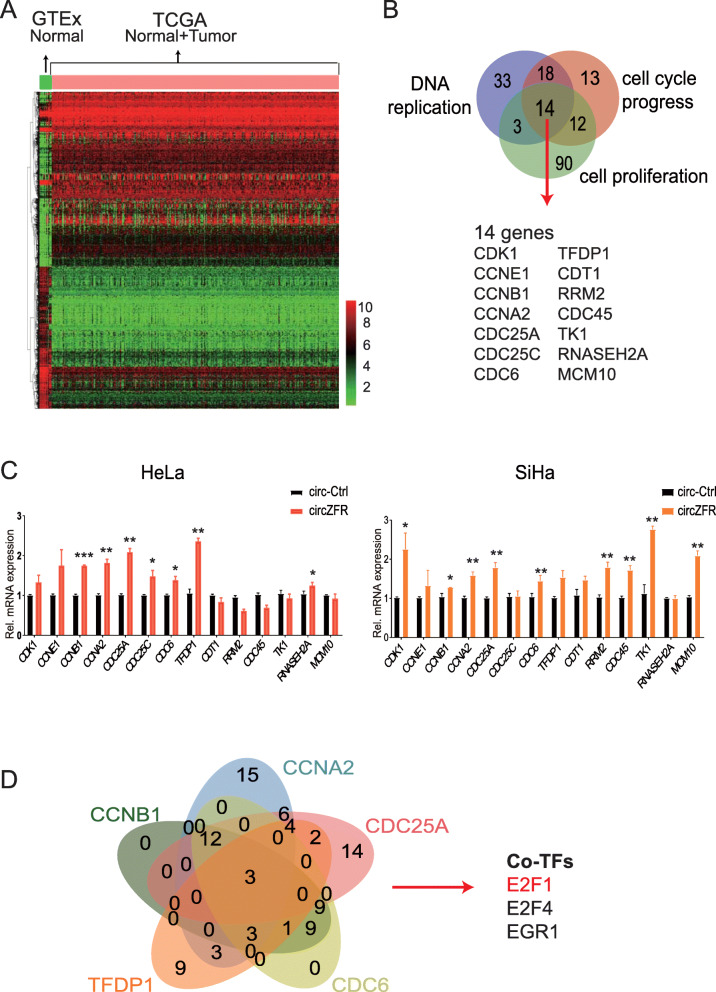
CircZFR activation of E2F1 pathway promoted the progression of cervical cancer. **a.** Heatmap showed the genes altered (Fold change > 2, *P* < 0.05) in cervical cancer compared with normal tissues according to TCGA and GTEx databases. Red refers to higher, and green refers to lower expression. **b.** Genes enrichment was performed using Cytoscape/BINGO software. A total of 14 upregulated genes in cervical cancer were enriched in the biological process of DNA replication, cell cycle progress, and cell proliferation. **c.** Relative mRNA expression of 14 candidate genes was detected by qRT-PCR in HeLa and SiHa cells stably overexpressing circZFR or circ-Ctrl. The mRNA expression of *CCNB1*, *CCNA2*, *CDC25A*, *CDC6*, and *TFDP1* was increased after the overexpression of circZFR. **d.** E2F1, E2F4 and EGR1 were identified as the transcription factors (TFs) of *CCNB1*, *CCNA2*, *CDC25A*, *CDC6*, and *TFDP1* using ChIPbase. *P< 0.05, **P< 0.01, ***P< 0.001

Next, we investigated whether circZFR could regulate cell proliferation and/or cell cycle progression. We analyzed the differentially expressed genes between cervical cancer (*n* = 306) and normal cervical tissues (*n* = 13) using two different databases from TCGA and GTEx. We identified 1266 differentially expressed mRNAs using cut-off criteria of a fold change > 2.0 and *P* < 0.05, of which 646 mRNAs were upregulated, and 620 were downregulated in cervical cancer (Fig. 4a). The Gene Ontology (GO) enrichment analysis demonstrated that the upregulated mRNAs were significantly enriched in DNA replication, positive regulation of cell cycle (G1/S phase transition), and cell proliferation (Fig. 4b and Fig. S2a-b). This suggested that these pathways may be critical to the pathogenesis of cervical cancer. We, therefore, hypothesize that CircZFR mainly promoted cell proliferation via inducing cell cycle G1/S phase transition. Therefore, we selected 14 upregulated mRNAs, which were enriched in DNA replication and cell cycle G1/S phase transition, and validated their expression through qRT-PCR analysis in HeLa and SiHa cells stably overexpressing circZFR. We found that the transcription level of *CCNB1*, *CCNA2*, *CDC25A*, *CDC6*, and *TFDP1* were significantly increased after the upregulation of circZFR compared to control (Fig. 4c). We further search for the transcription factors regulating these five genes in ChIPBase V2.0 (http://rna.sysu.edu.cn/chipbase/) and found shared transcription factors. The results showed that E2F1, E2F4, and EGR1 were co- transcription factors for *CCNB1*, *CCNA2*, *CDC25A*, *CDC6*, and *TFDP1* five genes (Fig. 4d). Moreover, Liu’s study validated E2F1 and E2F4 as the transcription factors for these five genes *CCNB1*, *CCNA2*, *CDC25A*, *CDC6*, and *TFDP1* by ChIP-Seq assay in HeLa cells [26]. Similarly, Gertz’s study validated EGR1 as the transcription factors for these five genes by ChIP-Seq assay in MCF7 cells [27]. The E2F1- and E2F4-binding peaks on CCNB1, CCNA2, CDC25A, CDC6, and TFDP1 transposable elements in HeLa cells were visualized using WashU Epigenome Browser (https://epigenomegateway.wustl.edu/) (Fig. S2c).

**Fig. 5:**
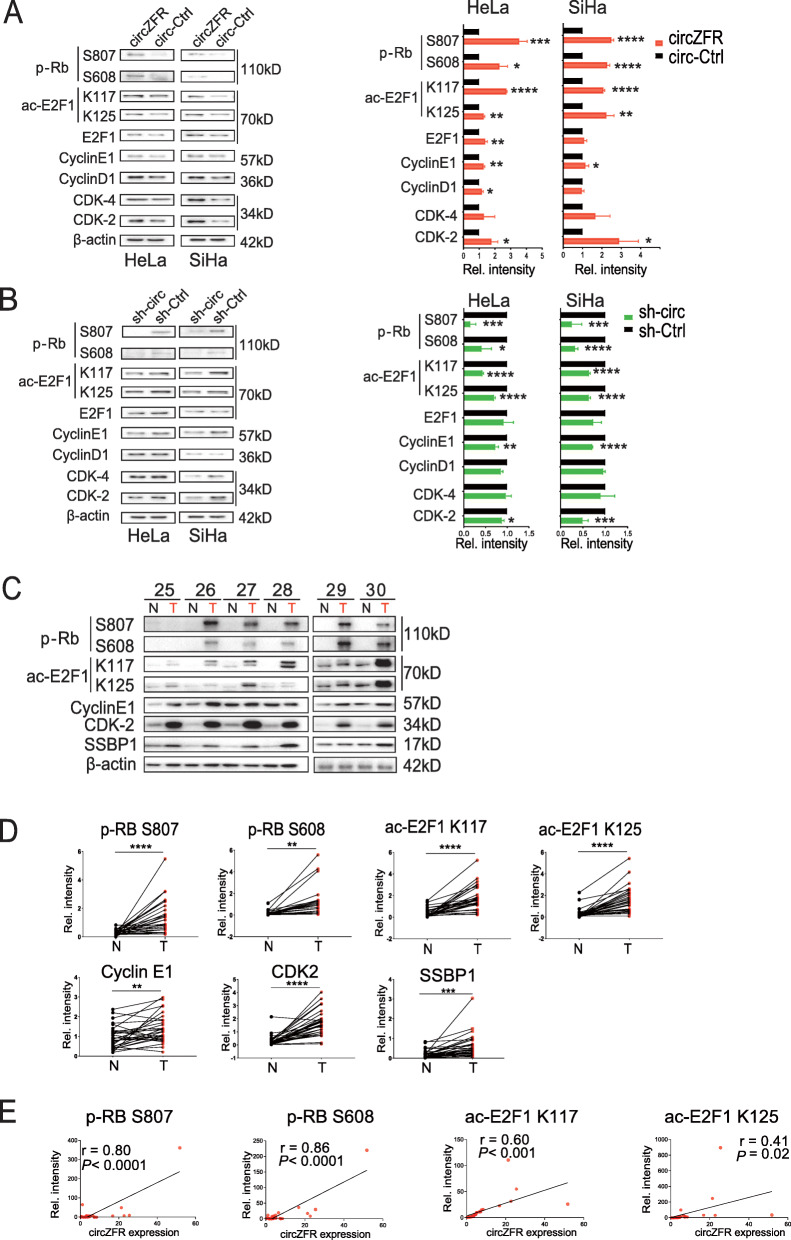
CircZFR induced Rb phosphorylation and E2F1 acetylation. **a-b.** p-Rb S608 and S807, ac-E2F1 K117 and K125, E2F1, cyclin-E1/−D1, and CDK4/2 protein levels were detected in HeLa and SiHa cells overexpressing circZFR, or circ-ZFR knockdown (sh-circ) and the corresponding controls (circ-Ctrl and sh-Ctrl, respectively). The relative intensities of these proteins were quantified by ImageJ (right panel). **c.** p-Rb S608 and S807, ac-E2F1 K117 and K125, cyclin-E1, and CDK2 were detected in tumors (T) and the paired normal tissues (N). **d.** The relative intensities of each protein in the paired dot plots of 30 cervical cancer patients were detected using ImageJ software. The analyses used a two-tailed paired Student’s t-test. **e.** The correlation between the expression of circZFR and the cell cycle proteins p-Rb and ac-E2F1. The Pearson correlation coefficients (r) and *p* values were performed using GraphPad Prism 7.0 software. *P< 0.05, **P< 0.01, ***P< 0.001, ****P< 0.0001

E2F1 acetylation has been demonstrated to confer active status for E2F1 [28] with acetylation sites on K117 and K125 [28]. We then verified that overexpression of circZFR significantly increased ac-E2F1 K117 and K125 acetylation, and conversely, knockdown of circZFR could significantly decrease ac-E2F1 K117 and K125 acetylation in HeLa and SiHa cells (Fig. 5a-b). To further study the biological effect of circZFR on E2F1 acetylation in vivo, we compared the E2F1 acetylation status in 30 cervical tumors compared to adjacent normal tissues. We found that ac-E2F1 K117 and K125 acetylation were elevated in cervical cancer compared with normal tissues (Fig. 5c-d and S3a). The correlation analyses revealed the positive relationship between circZFR and high acetylation of ac-E2F1 K117, and K125 (Fig. 5e).

### CircZFR induced Rb phosphorylation by promoting CDK2/cyclin E1 and CDK2/SSBP1 assembly

Phosphorylation regulates Rb interactions with other proteins. Hyperphosphorylation of Rb by CDK2/Cyclin E1 complex was demonstrated to release E2F1 and promote E2F1 acetylation, allowing the transcription of genes required for the G1-S phase transition and DNA replication [28, 29].

To investigate the mechanism by which circZFR regulates E2F1 acetylation, we tested several phosphorylation sites of Rb (S807, S608, S780, and T821). We identified that overexpression of circZFR significantly increased p-Rb S807 and S608 phosphorylation, while knockdown of circZFR expression significantly decreased p-Rb S807 and S608 phosphorylation (Fig. 5a-b). However, circZFR could not affect p-Rb S780 or T821 phosphorylation (Fig. S3b). Furthermore, the tissue lysates also suggested that phosphorylation of p-Rb S807 and S608 was elevated in cervical cancer tissues compared to adjacent normal tissues (Fig. 5c-d and S3a). Correlation analyses revealed a positive relationship between circZFR and p-Rb S807 and S608 phosphorylation (Fig. 5e). We also validated the phosphorylation of p-Rb S807 and S608 in cervical cancer tissues and adjacent normal tissues using immunohistochemistry and the results were consistent with western blot (Fig. S3c).

We further investigated the mechanism of circZFR regulation of p-Rb S807 and S608 phosphorylation. Considering that several cyclin/CDK families have been demonstrated to regulate p-Rb phosphorylation, we tested the protein expression of CDK4, CDK2, cyclin D1, and cyclin E1. We found that overexpression of circZFR significantly increased CDK2 and cyclin E1 expression, while knockdown of circZFR significantly decreased both proteins (Fig. 5a-b). Besides, the expression of CDK2 and cyclin E1 increased in cervical cancer compared with adjacent normal tissues (Fig. 5c-d and S3a). Correlation analyses revealed the positive relationship between circZFR and expression of cyclin E1 (Fig. 5e).

**Fig. 6:**
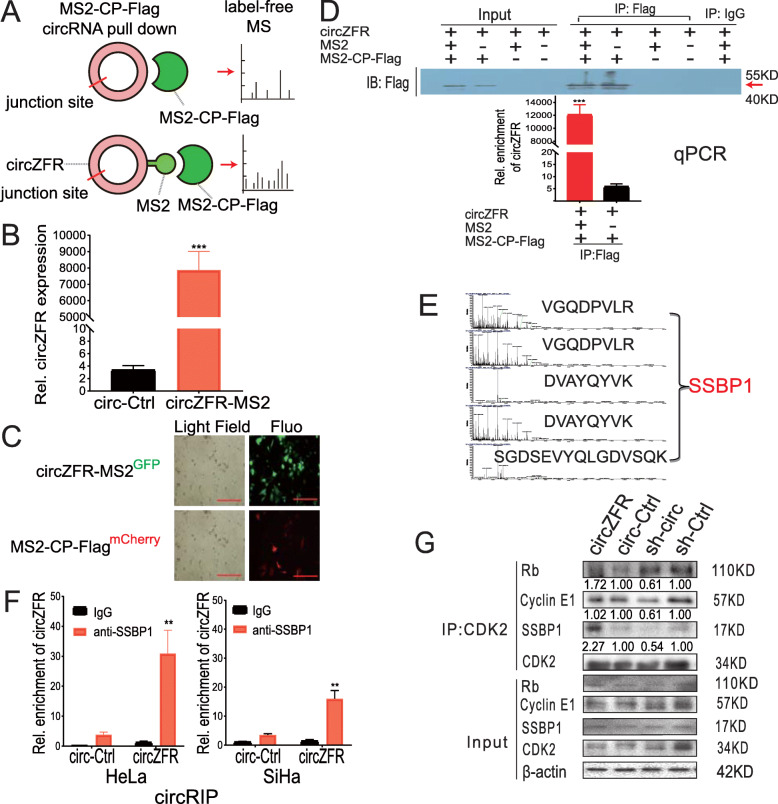
CircZFR promotes CDK2/cyclin-E1 and CDK2/SSBP1 assembly and interacts with SSBP1. **a**. Model of i**n vivo circRNA pull-down** using MS2-tagging system, and the following label-free mass spectrometric (MS) analysis. **b**. Confirmation of the overexpression of circZFR using qRT-PCR analysis. **c**. Co-transfection of circZFR-MS2^GFP^ and MS2-CP-Flag^mCherry^ plasmids to induce the expression of MS2 RNA hairpins with overexpressed circZFR and a fusion protein MS2-CP-Flag, which could recognize MS2 RNA hairpins (Scale bar = 200 μm). The green fluorescence-labeled circZFR (up) and the red fluorescence-labeled MS2-CP-Flag (bottom). **d**. Western Blot test the MS2-CP-Flag pulled down by anti-Flag (up). The enrichment of circZFR in the complex with MS2-CP-Flag was detected by qRT-PCR (bottom). The assays were done twice in triplicate. **e**. The protein complex with MS2-CP-Flag was tested using a label-free MS. The peptides were matched to SSBP1. **f**. circRNA immunoprecipitation (circRIP) assay to measure the amount of circZFR pulled down by SSBP1 and IgG antibodies in the HeLa and SiHa cells stably overexpressed circZFR and circ-Ctrl. The assays were done twice in triplicate. **g**. The lysates prepared from the HeLa cells stably overexpressing circZFR, or circZFR knockdown (sh-circ) and the corresponding controls (circ-Ctrl, and sh-Ctrl, respectively) were subjected to immunoprecipitation using anti-CDK2. **P< 0.001, ****P< 0.0001, MS2-CP: MS2 bacteriophage coat protein, MS: mass spectrometric, GFP: green fluorescent protein, circRIP: circRNA immunoprecipitation

To identify the protein partner of circZFR, we performed a proteomic screen with MS2-CP-Flag circRNAs pull-down assay (Fig. 6a). First, the overexpression of circZFR in the circZFR-MS2, tagging system was confirmed using qRT-PCR analysis (Fig. 6b). Sanger sequencing confirmed the junction site, which indicated that this design did not impair the circularization of circZFR (Fig. S1c). We constructed two plasmids expressing circZFR-MS2^GFP^ and MS2-CP-Flag^mCherry^, respectively. Next, we further validated our RIP assay using the circZFR-MS2 tagging system into HeLa cells through the co-transfection of circZFR and MS2-CP-Flag (Fig. 6c). The protein complexes associated between MS2 and MS2-CP were pulled down using Flag antibodies. We detected the capture protein MS2-CP-Flag using western blot analysis with anti-Flag antibodies (Fig. 6d, top panel). We validated that circZFR was highly enriched following capture using qRT-PCR analysis (Fig. 6d, bottom panel), which demonstrated the specificity of our pull-down isolation. Finally, through a label-free MS analysis, we detected the peptides of proteins bound with the circZFR, and matched all four peptides to SSBP1 (Fig. 6e). These data validated the interaction between circZFR with SSBP1. To further verify the binding of circZFR to SSBP1, a circRIP assay was conducted in HeLa and SiHa cells using anti-SSBP1 antibodies. Increases of circZFR pull-down were enriched after anti-SSBP1 immunoprecipitation compared with IgG (Fig. 6f).

The dynamic regulation of p-Rb phosphorylation by the cyclin/CDK complex could promote cells to exit the G0 phase, traverse G1, and enter the S phase [30, 31]. The initiation of p-Rb phosphorylation was suggested to be associated with CDK4/Cyclin D activation, and hyperphosphorylation of Rb during the late G1 phase required CDK2/cyclin E activation. It is possible that CDK2/Cyclin A complex could promote Rb phosphorylation during the S phase [30–32]. Because we found that circZFR induced phosphorylation of p-Rb S807 and S608, and Rb phosphorylation could be regulated by CDK2/cyclin E1 complex, we further investigated the regulation of CDK2/cyclin E1 complex by circZFR using a Co-IP assay. We found that circZFR overexpression enhanced the formation of CDK2/Rb, CDK2/Cyclin E1, and CDK2/SSBP1 complexes and, inversely, knockdown of circZFR inhibited the formation of the same complexes (Fig. 6g). Thus, we demonstrated that the overexpression of circZFR could serve as a protein partner or docking site molecule to enhance the assembly of SSBP1 and CDK2 complex, and inversely, circZFR knockdown could dissociate SSBP1 and CDK2 complex.

Furthermore, we knocked down SSBP1 in HeLa and SiHa cells stably overexpressing circZFR (Fig. S4a). We chose sh-SSBP1#1 for the following assays because it had the highest efficiency of interference. CCK-8 assay and transwell matrigel migration and invasion assays showed that reduced SSBP1 expression by shRNA inhibited cell proliferation, migration, and invasion in stably circZFR overexpressing cells (HeLa and SiHa cells) (Fig. S4b-d). We found that knockdown of SSBP1 expression significantly decreased p-Rb (S807 and S608 phosphorylation) and ac-E2F1 (K117 and K125 acetylation) expression (Fig. S4e). Moreover, the transcription of *CCNB1*, *CCNA2*, *CDC25A*, *CDC6*, and *TFDP1* were inhibited after knocking down SSBP1 (Fig. S4f). We also knocked down E2F1 in circZFR stably overexpressed HeLa cells with two different siRNAs (si-E2F1#1 and si-E2F1#2) (Fig. S5a), but the cell proliferation was not inhibited (Fig. S5b).

### CircZFR regulated the growth of cervical cancer cells in vivo

**Fig. 7:**
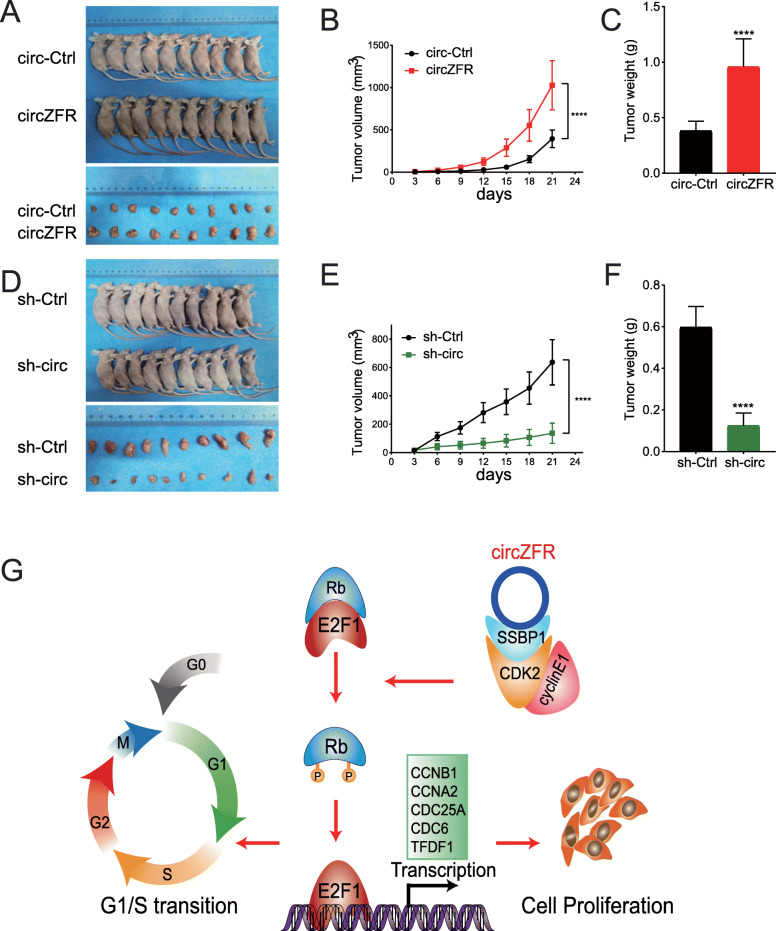
CircZFR promotes the growth of cervical cancer cells in vivo. **a.** Nude female mice were subcutaneously injected with 2 × 10^7^ stably overexpressed circZFR and the corresponding control HeLa cells (*n* = 10 for each group), and the tumors were extracted after 21 days. **b.** The volume of tumors in figure a was measured every 3 days. **c.** The weight of tumors in figure a was measured. **d.** Nude female mice were subcutaneously injected with 2 × 10^7^ stably knockdown circZFR and the corresponding control HeLa cells (n = 10 for each group), and the tumors were extracted after 21 days. **e.** The volume of tumors in figure d was measured every 3 days. **f.** The weight of tumors in figure d was measured. **g.** Model of circZFR-mediated p-Rb phosphorylation promoting the G1/S transition and cell proliferation in cervical cancer. ****P< 0.0001

We subcutaneously injected stably overexpressed, knockdown circZFR and the corresponding control HeLa cells into nude mice. After 21 days of observation, the results showed that circZFR significantly promoted tumor growth (Fig. 7a-c). And reducing circZFR inhibited the tumor growth (Fig. 7d-f).

Finally, our working model illustrates how circZFR could recruit SSBP1, which acts as a scaffold protein for the CDK2/Cyclin E1 complex (Fig. 7g). The activated complex phosphorylates p-Rb S807 and S608, which interrupts p-Rb-E2F1 pairing. The released E2F1 transcription factor from p-Rb triggers the transcription of target DNA replication-associated genes to promote the G1/S transition thus allows cervical cancer cell proliferation (Fig. 7g).

## Discussion

In this study, we identified that circZFR was the most significantly upregulated circRNAs in cervical cancer cells and tissues. CircZFR acted as an activator of Rb-E2F1 signaling, which promoted the G1/S transition, cell proliferation, migration, and invasion of squamous cervical cancer. CircZFR phosphorylated Rb by regulating the SSBP1/CDK2/cyclin E1 complexes. Furthermore, we verified that circZFR promoted the assembly and activation of CDK2/cyclin E1 complexes through binding with SSBP1, which acted as a protein partner.

There is emerging appreciation that ncRNA, such as long non-coding RNA (lncRNA), miRNA, and circRNA, play critical roles in the screening cancer, prediction treatment response, and prognosis of cancer [33, 34]. The evidence supporting circRNAs regulating the cell cycle of tumor cells is still limited. The importance of cirRNAs in cervical cancer has been described recently. Ou et al. reported that circRNA-AKT1 promotes cervical cancer progression through sequestering miR-942-5p [35]. Ji et al. reported that circSLC26A4 regulated miR-1287-5p/HOXA7 and accelerated cervical cancer progression [36]. However, the studies describing the precise role of circZFR regulating cell proliferation and cell cycle are limited. Yang et al. reported that circZFR promoted hepatocellular carcinoma cell proliferation through regulating miR-522/AKT1 [37]. Li et al. reported that the growth of non-small-cell lung cancer could be inhibited after interfering circZFR [38]. But the effect of circZFR in cervical cancer has not been reported. In this study, we found that the overexpression of circZFR in cervical cancer was associated with lymphatic metastasis, high proliferation state and elevation of biomarker SCC Ag value. Although the recurrent rate of the involved patients in this study is only 2.5%, with progression-free survival of 8.17 months and no death case, longer follow-up was needed to investigate the prognostic role of circZFR.

DNA replication, G1/S transition, and cell proliferation were demonstrated to be an essential driving pathway for cervical cancer, with E2F1 representing the core transcription factor for regulating these pathways. Rb-E2F axis is a critical pathway regulating the pathogenesis and progression of cervical cancer [39]. Aberrant Rb-E2F signaling could lead to the irreversible G1/S switch in the activation of growth factors [40]. Rb is encoded by the tumor suppressor retinoblastoma gene (*RB1*), which can arrest the cell cycle in its active hypophosphorylated form. The tumor suppression function of Rb is characterized as the interaction between Rb and the E2F transactivation domain (E2F^TD^) [41, 42]. As a member of the pocket protein complex, Rb can be phosphorylated by multiple CDK sites [43–47]. The interaction between the pocket domain in Rb and E2F^TD^ is inhibited by S608 phosphorylation [48, 49]. Besides, according to the present evidence, p-Rb S807 phosphorylation could prime the phosphorylation at another site and might be critical for the Rb phosphorylation [47]. Here, we found overexpression or silence of circZFR separately increased or decreased p-Rb S807 and S608 phosphorylation ac-E2F1 K117 and K125 acetylation in cervical cancer cells. Moreover, we demonstrate overexpression of circZFR promoted the p-Rb S807 and S608 phosphorylation and ac-E2F1 K117 and K125 acetylation using paired cervical cancer and normal tissues, which suggested that circZFR is a central upstream regulator in the activation of Rb-E2F1 signaling.

With the development and usage of high-throughput screening, more than 1000 RBPs have been identified, and multiple RBPs were demonstrated to be associated with carcinogenesis [10, 11]. But the function of most RBPs remains unknown. The reports about circRNAs modulating the pathogenesis of malignant tumors via targeting of RBPs are limited. Circ-ACC1 has been demonstrated to directly bind to AMPK β and γ subunits and facilitate AMPK holoenzyme assembly, stability, and activity [50]. CircRHOT1 was found to promote hepatocellular carcinoma (HCC) growth through the recruitment of TIP60 to the promoter of *NR2F6* and initiating *NR2F6* transcription [51]. A study reported that circZKSCAN1 physically bound an RBP FMRP against CCAR1 complex and inhibited HCC [52]. Circ-Foxo3 was found to bind both cyclin-dependent kinase (CDK) 2 and a known CDK-inhibitor p21. The ternary complex of circ-Foxo3-p21-CDK2 hijacked CDK2 and disturbed the formation of the CDK2/cyclin E1 complex, arresting the cell cycle in G1 phase [53]. Quaking (QKI), as an RBP with the K-homology (KH) RNA binding domain, could promote circRNAs production through binding sites in introns [54]. There is no report about the interaction between circRNA and RBP in cervical cancer. Through circRIP, and RNA pull-down assays/RIP, we uncovered that upregulation of circZFR promotes circZFR binding with SSBP1 as well as SSBP1/CDK2/cyclin E1 complex assembly. This regulation could be further inhibited through the downregulating of circZFR. This suggested that SSBP1 be a candidate RBP assisting SSBP1/CDK2/cyclin E1 assembly. The cell proliferation, migration, and invasion induced by circZFR were inhibited after knocking down SSBP1. Moreover, the inhibition of SSBP1 also decreased p-Rb S807 and S608 phosphorylation and E2F1 K117 and K125 acetylation. However, this rescue was not observed after knocking down E2F1, which suggested that SSBP1 was the critical point for circZFR promoting cervical cancer cell growth.

There still exists limitations in this study. We could not get ac-E2F1 K117 and K125 antibodies with the application for immunohistochemistry. The ac-E2F1 acetylation (K117 and K125) and p-Rb phosphorylation (S608 and S807) require a large size of samples to validate in the future.

## Conclusions

In summary, the results of this study identified that circZFR was upregulated in cervical cancer. CircZFR could promote cervical cancer cell proliferation, migration, and invasion through the induction of p-Rb S807 and S608 phosphorylation and activating E2F1 signaling. Furthermore, we provided conclusive evidence of a binding of circZFR and SSBP1, which acted as a scaffolding protein in the assembly and activation of SSBP1/CDK2/cyclin E1 complexes. As a novel positive regulator of E2F1 signaling, circZFR may be a potential circulating biomarker with implications for the detection of cervical cancer.

## Supplementary Information

**Additional file 1: Fig. S1.** a. Flow of the screening and selection of circZFR. b. Verification of qPCR products of specific primers for circZFR. c. Sanger sequencing confirmed the junction site of circZFR (Exon 13 and 17) in the circZFR-MS2 plasmid.

**Additional file 2: Fig. S2.** a-b. GO enrichment analysis of upregulated genes in cervical cancer. **c.** E2F1- and E2F4-binding peaks on CCNB1, CCNA2, CDC25A, CDC6, and TFDP1 transposable elements in HeLa cells are visualized using WashU Epigenome Browser.

**Additional file 3: Fig. S3.** a. Western blot analysis showing the correlation between the expression of circZFR, the cell cycle proteins p-Rb S608 and S807, ac-E2F1, cyclin-E1, CDK2, and SSBP1. Proteins were detected in 30 tumors (T) and the paired normal tissues (N). The relative intensities of each protein in the paired dot plots were detected using ImageJ software (bottom). The analyses used a two-tailed paired Student’s t-test. b. p-Rb T821 and S780 protein levels were detected in HeLa and SiHa cells overexpressing circZFR, or circ-ZFR knockdown (sh-circ) and the corresponding controls (circ-Ctrl and sh-Ctrl, respectively). c. Immunohistochemistry of p-Rb S807 and S608 in 30 tumors (T) and the paired normal tissues (N). Left showed a representative image, and the right panel showed statistics analysis. **P< 0.01, ***P< 0.001, ****P < 0.0001.

**Additional file 4: Fig. S4.** Downregulation of SSBP1 inhibited proliferation, migration, and invasion of cervical cancer cells and inhibited Rb phosphorylation and E2F1 acetylation. a. qRT-PCR analysis of SSBP1 expression in HeLa and SiHa cells after transfection using three different shRNAs targeting SSBP1 or control-shRNA (sh-Ctrl). Sh-SSBP1#1 showed the highest knockdown efficacy. b. Downregulation of SSBP1 inhibited the proliferation ability of HeLa and SiHa cells as measured by CCK-8 assay. c-d. Knockdown of SSBP1 impeded cell migration and invasion ability of HeLa and SiHa cells as measured by transwell matrigel migration and invasion assays (Scale bar in c and d = 50 μm). e. p-Rb S608 and S807, ac-E2F1 K117 and K125, and SSBP1 protein levels were detected in HeLa and SiHa cells knocking down SSBP1 (sh-SSBP1#1) and the corresponding controls (sh-Ctrl). The relative intensities of these proteins were quantified by ImageJ (right panel). f. The mRNA expression of *CCNB1*, *CCNA2*, *CDC25A*, *CDC6*, and *TFDP1* was decreased after knocking down SSBP1. *P < 0.05, **P< 0.01, ***P< 0.001, ****P< 0.0001.

**Additional file 5: Fig. S5.** Downregulation of E2F1 did not inhibit cervical cancer cell proliferation. a. qRT-PCR analysis of E2F1 expression in HeLa cells after transfection using two different siRNAs targeting E2F1 (si-E2F1#1 and si-E2F1#2) or control-siRNA (ctrl). b. Downregulation of E2F1 did not inhibit the proliferation ability of circZFR stably overexpressed HeLa cells as measured by CCK-8 assay.

**Additional file 6: Supplementary Table 1.** Primers used in qRT-PCR analysis. **Supplementary Table 2.** Sequence of circZFR shRNAs and negative control. **Supplementary Table 3.** Target sequences of SSBP1 shRNAs. **Supplementary Table 4.** Sequences of E2F1 siRNAs.

## Data Availability

The datasets supporting the conclusions of this article are included within the article and its Additional files.

## Abbreviations

circRNA: Circular RNA
ceRNA: Competitive endogenous RNA
EdU: 5-Ethynyl-20-deoxyuridine
GO: Gene Ontology
HPV: Human papilloma virus
KH: K-homology
LCHI: Liaoning Cancer Hospital & Institute
miRNA: microRNA
MS2-CP: MS2 bacteriophage coat protein
ncRNA: Non-coding RNAs
QKI: Quaking
RBDs: RNA-binding domains
RBPs: RNA-binding proteins
SCC Ag: Squamous cell carcinoma antigen
SSBP1: Single-Stranded DNA Binding Protein 1

## References

[1] Siegel RL, Miller KD, Jemal A (2020). Cancer statistics, 2020. CA Cancer J Clin.

[2] Lei J, Andrae B, Ploner A, Lagheden C, Eklund C, Nordqvist Kleppe S, Wang J, Fang F, Dillner J, Elfstrom KM, Sparen P (2019). Cervical screening and risk of adenosquamous and rare histological types of invasive cervical carcinoma: population based nested case-control study. BMJ.

[3] Canfell K, Kim JJ, Brisson M, Keane A, Simms KT, Caruana M, Burger EA, Martin D, Nguyen DTN, Benard E (2020). Mortality impact of achieving WHO cervical cancer elimination targets: a comparative modelling analysis in 78 low-income and lower-middle-income countries. Lancet.

[4] Fidler MM, Gupta S, Soerjomataram I, Ferlay J, Steliarova-Foucher E, Bray F (2017). Cancer incidence and mortality among young adults aged 20-39 years worldwide in 2012: a population-based study. Lancet Oncol.

[5] Slack FJ, Chinnaiyan AM (2019). The role of non-coding RNAs in oncology. Cell.

[6] Kristensen LS, Andersen MS, Stagsted LVW, Ebbesen KK, Hansen TB, Kjems J (2019). The biogenesis, biology and characterization of circular RNAs. Nat Rev Genet.

[7] Chen LL (2016). The biogenesis and emerging roles of circular RNAs. Nat Rev Mol Cell Biol.

[8] Nigro JM, Cho KR, Fearon ER, Kern SE, Ruppert JM, Oliner JD, Kinzler KW, Vogelstein B (1991). Scrambled exons. Cell.

[9] Hansen TB, Jensen TI, Clausen BH, Bramsen JB, Finsen B, Damgaard CK, Kjems J (2013). Natural RNA circles function as efficient microRNA sponges. Nature.

[10] Castello A, Fischer B, Eichelbaum K, Horos R, Beckmann BM, Strein C, Davey NE, Humphreys DT, Preiss T, Steinmetz LM (2012). Insights into RNA biology from an atlas of mammalian mRNA-binding proteins. Cell.

[11] Wang ZL, Li B, Luo YX, Lin Q, Liu SR, Zhang XQ, Zhou H, Yang JH, Qu LH (2018). Comprehensive genomic characterization of RNA-binding proteins across human cancers. Cell Rep.

[12] Jankowsky E, Harris ME (2015). Specificity and nonspecificity in RNA-protein interactions. Nat Rev Mol Cell Biol.

[13] Lunde BM, Moore C, Varani G (2007). RNA-binding proteins: modular design for efficient function. Nat Rev Mol Cell Biol.

[14] Chuang TJ, Wu CS, Chen CY, Hung LY, Chiang TW, Yang MY (2016). NCLscan: accurate identification of non-co-linear transcripts (fusion, trans-splicing and circular RNA) with a good balance between sensitivity and precision. Nucleic Acids Res.

[15] Enuka Y, Lauriola M, Feldman ME, Sas-Chen A, Ulitsky I, Yarden Y (2016). Circular RNAs are long-lived and display only minimal early alterations in response to a growth factor. Nucleic Acids Res.

[16] Rybak-Wolf A, Stottmeister C, Glazar P, Jens M, Pino N, Giusti S, Hanan M, Behm M, Bartok O, Ashwal-Fluss R (2015). Circular RNAs in the mammalian brain are highly abundant, conserved, and dynamically expressed. Mol Cell.

[17] Wang S, Pan Y, Zhang R, Xu T, Wu W, Zhang R, Wang C, Huang H, Calin CA, Yang H, Claret FX (2016). Hsa-miR-24-3p increases nasopharyngeal carcinoma radiosensitivity by targeting both the 3'UTR and 5'UTR of Jab1/CSN5. Oncogene.

[18] Yuan JH, Liu XN, Wang TT, Pan W, Tao QF, Zhou WP, Wang F, Sun SH (2017). The MBNL3 splicing factor promotes hepatocellular carcinoma by increasing PXN expression through the alternative splicing of lncRNA-PXN-AS1. Nat Cell Biol.

[19] Pan Y, Zhang Q, Tian L, Wang X, Fan X, Zhang H, Claret FX, Yang H (2012). Jab1/CSN5 negatively regulates p27 and plays a role in the pathogenesis of nasopharyngeal carcinoma. Cancer Res.

[20] Pan Y, Wang S, Su B, Zhou F, Zhang R, Xu T, Zhang R, Leventaki V, Drakos E, Liu W, Claret FX (2017). Stat3 contributes to Cancer progression by regulating Jab1/Csn5 expression. Oncogene.

[21] Pan Y, Zhang Q, Atsaves V, Yang H, Claret FX (2013). Suppression of Jab1/CSN5 induces radio- and chemo-sensitivity in nasopharyngeal carcinoma through changes to the DNA damage and repair pathways. Oncogene.

[22] Bertrand E, Chartrand P, Schaefer M, Shenoy SM, Singer RH, Long RM (1998). Localization of ASH1 mRNA particles in living yeast. Mol Cell.

[23] Holdt LM, Stahringer A, Sass K, Pichler G, Kulak NA, Wilfert W, Kohlmaier A, Herbst A, Northoff BH, Nicolaou A (2016). Circular non-coding RNA ANRIL modulates ribosomal RNA maturation and atherosclerosis in humans. Nat Commun.

[24] Liu J, Wang D, Long Z, Liu J, Li W (2018). CircRNA8924 promotes cervical Cancer cell proliferation, migration and invasion by competitively binding to MiR-518d-5p /519-5p family and modulating the expression of CBX8. Cell Physiol Biochem.

[25] Jiao J, Zhang T, Jiao X, Huang T, Zhao L, Ma D, Cui B (2020). hsa_circ_0000745 promotes cervical cancer by increasing cell proliferation, migration, and invasion. J Cell Physiol.

[26] Liu W, Tanasa B, Tyurina OV, Zhou TY, Gassmann R, Liu WT, Ohgi KA, Benner C, Garcia-Bassets I, Aggarwal AK (2010). PHF8 mediates histone H4 lysine 20 Demethylation events involved in cell cycle progression. Nature.

[27] Gertz J, Savic D, Varley KE, Partridge EC, Safi A, Jain P, Cooper GM, Reddy TE, Crawford GE, Myers RM. Distinct properties of cell type-specific and shared transcription factor binding sites. Mol Cell. 2013;52:25–36. 10.1016/j.molcel.2013.08.037 PMC3811135 24076218

[28] Martinez-Balbas MA, Bauer UM, Nielsen SJ, Brehm A, Kouzarides T (2000). Regulation of E2F1 activity by acetylation. EMBO J.

[29] Harbour JW, Dean DC (2000). The Rb/E2F pathway: expanding roles and emerging paradigms. Genes Dev.

[30] DeCaprio JA, Furukawa Y, Ajchenbaum F, Griffin JD, Livingston DM (1992). The retinoblastoma-susceptibility gene product becomes phosphorylated in multiple stages during cell cycle entry and progression. Proc Natl Acad Sci U S A.

[31] Mittnacht S (1998). Control of pRB phosphorylation. Curr Opin Genet Dev.

[32] Kaye FJ, Kratzke RA, Gerster JL, Horowitz JM (1990). A single amino acid substitution results in a retinoblastoma protein defective in phosphorylation and oncoprotein binding. Proc Natl Acad Sci U S A.

[33] Kopp F, Mendell JT (2018). Functional classification and experimental dissection of Long noncoding RNAs. Cell.

[34] Rupaimoole R, Slack FJ (2017). MicroRNA therapeutics: towards a new era for the management of cancer and other diseases. Nat Rev Drug Discov.

[35] Ou R, Mo L, Tang H, Leng S, Zhu H, Zhao L, Ren Y, Xu Y (2020). circRNA-AKT1 sequesters miR-942-5p to Upregulate AKT1 and promote cervical Cancer progression. Mol Ther Nucleic Acids.

[36] Ji F, Du R, Chen T, Zhang M, Zhu Y, Luo X, Ding Y (2020). Circular RNA circSLC26A4 accelerates cervical Cancer progression via miR-1287-5p/HOXA7 Axis. Mol Ther Nucleic Acids.

[37] Yang X, Liu L, Zou H, Zheng YW, Wang KP (2019). circZFR promotes cell proliferation and migration by regulating miR-511/AKT1 axis in hepatocellular carcinoma. Dig Liver Dis.

[38] Li H, Liu F, Qin W (2020). Circ_0072083 interference enhances growth-inhibiting effects of cisplatin in non-small-cell lung cancer cells via miR-545-3p/CBLL1 axis. Cancer Cell Int.

[39] Burkhart DL, Sage J (2008). Cellular mechanisms of tumour suppression by the retinoblastoma gene. Nat Rev Cancer.

[40] Yao G, Lee TJ, Mori S, Nevins JR, You L (2008). A bistable Rb-E2F switch underlies the restriction point. Nat Cell Biol.

[41] Lee C, Chang JH, Lee HS, Cho Y (2002). Structural basis for the recognition of the E2F transactivation domain by the retinoblastoma tumor suppressor. Genes Dev.

[42] Xiao B, Spencer J, Clements A, Ali-Khan N, Mittnacht S, Broceno C, Burghammer M, Perrakis A, Marmorstein R, Gamblin SJ (2003). Crystal structure of the retinoblastoma tumor suppressor protein bound to E2F and the molecular basis of its regulation. Proc Natl Acad Sci U S A.

[43] Adams PD, Li X, Sellers WR, Baker KB, Leng X, Harper JW, Taya Y, Kaelin WG (1999). Retinoblastoma protein contains a C-terminal motif that targets it for phosphorylation by cyclin-cdk complexes. Mol Cell Biol.

[44] Schulman BA, Lindstrom DL, Harlow E (1998). Substrate recruitment to cyclin-dependent kinase 2 by a multipurpose docking site on cyclin a. Proc Natl Acad Sci U S A.

[45] Zarkowska T, Mittnacht S (1997). Differential phosphorylation of the retinoblastoma protein by G1/S cyclin-dependent kinases. J Biol Chem.

[46] Knudsen ES, Wang JY (1996). Differential regulation of retinoblastoma protein function by specific Cdk phosphorylation sites. J Biol Chem.

[47] Rubin SM (2013). Deciphering the retinoblastoma protein phosphorylation code. Trends Biochem Sci.

[48] Burke JR, Hura GL, Rubin SM (2012). Structures of inactive retinoblastoma protein reveal multiple mechanisms for cell cycle control. Genes Dev.

[49] Burke JR, Deshong AJ, Pelton JG, Rubin SM (2010). Phosphorylation-induced conformational changes in the retinoblastoma protein inhibit E2F transactivation domain binding. J Biol Chem.

[50] Li Q, Wang Y, Wu S, Zhou Z, Ding X, Shi R, Thorne RF, Zhang XD, Hu W, Wu M (2019). CircACC1 regulates assembly and activation of AMPK complex under metabolic stress. Cell Metab.

[51] Wang L, Long H, Zheng Q, Bo X, Xiao X, Li B (2019). Circular RNA circRHOT1 promotes hepatocellular carcinoma progression by initiation of NR2F6 expression. Mol Cancer.

[52] Zhu YJ, Zheng B, Luo GJ, Ma XK, Lu XY, Lin XM, Yang S, Zhao Q, Wu T, Li ZX (2019). Circular RNAs negatively regulate cancer stem cells by physically binding FMRP against CCAR1 complex in hepatocellular carcinoma. Theranostics.

[53] Du WW, Yang W, Liu E, Yang Z, Dhaliwal P, Yang BB (2016). Foxo3 circular RNA retards cell cycle progression via forming ternary complexes with p21 and CDK2. Nucleic Acids Res.

[54] Conn SJ, Pillman KA, Toubia J, Conn VM, Salmanidis M, Phillips CA, Roslan S, Schreiber AW, Gregory PA, Goodall GJ (2015). The RNA binding protein quaking regulates formation of circRNAs. Cell.

