# Supplementary figures and images for: The circular RNA circZFR phosphorylates Rb promoting cervical cancer progression by regulating the SSBP1/CDK2/cyclin E1 complex
Source: J Exp Clin Cancer Res. 2021 Jan 30;40:48. doi: 10.1186/s13046-021-01849-2 (PMC7846991; doi:10.1186/s13046-021-01849-2)

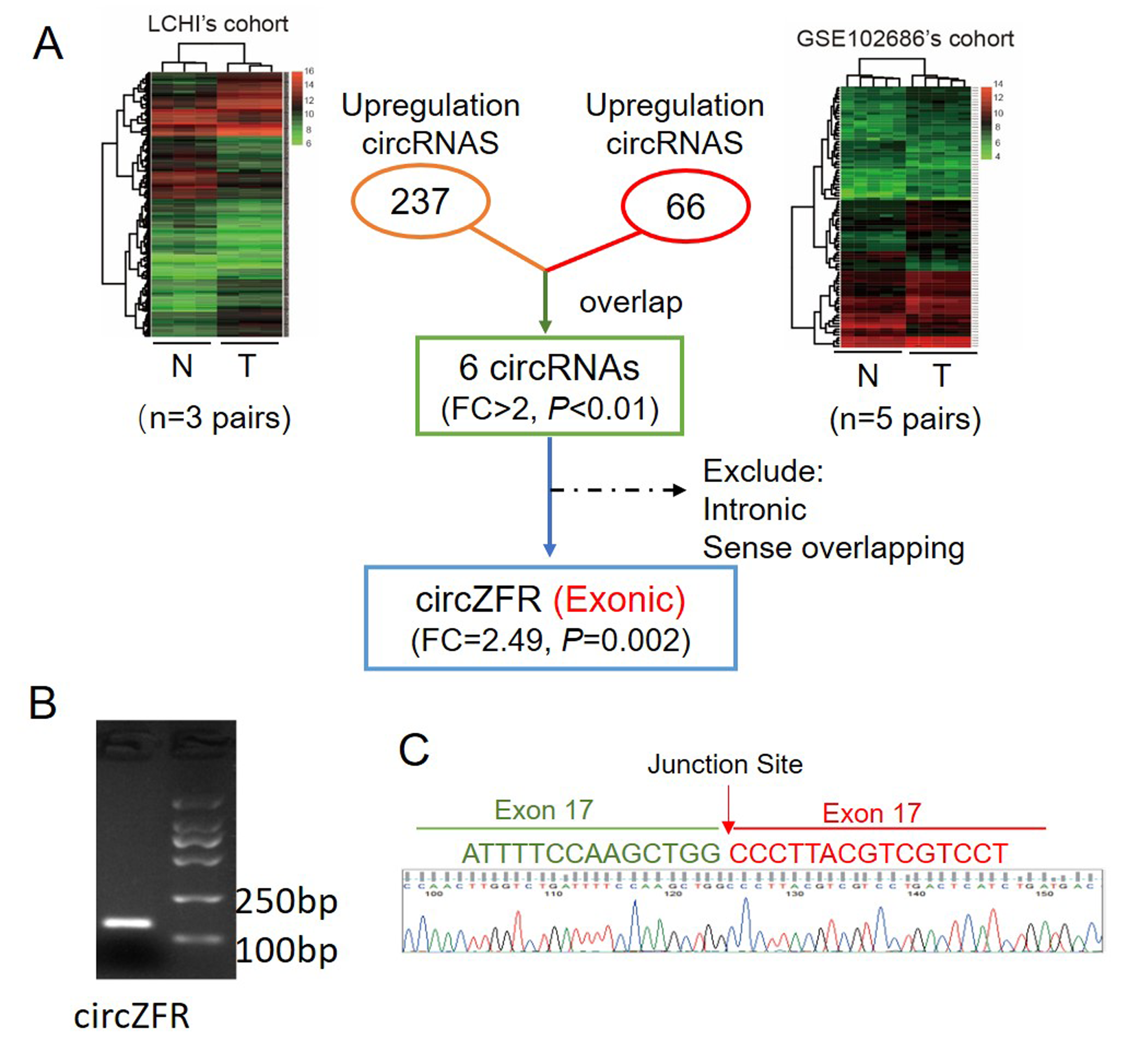

Supplement: Supplementary file 1 — Additional file 1: Fig. S1. a. Flow of the screening and selection of circZFR. b. Verification of qPCR products of specific primers for circZFR. c. Sanger sequencing confirmed the junction site of circZFR (Exon 13 and 17) in the circZFR-MS2 plasmid. [file 13046_2021_1849_MOESM1_ESM.tif]

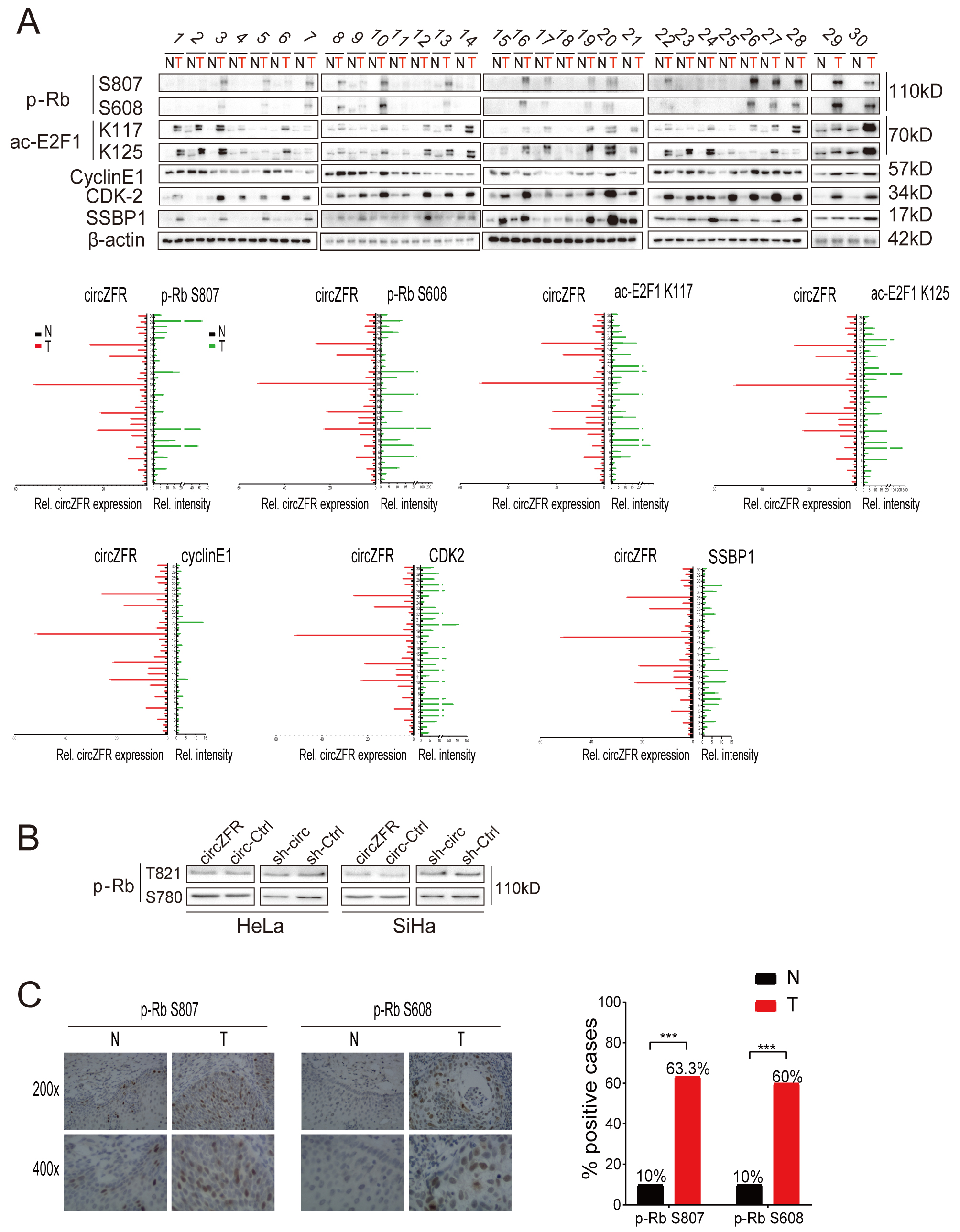

Supplement: Supplementary file 3 — Additional file 3: Fig. S3. a. Western blot analysis showing the correlation between the expression of circZFR, the cell cycle proteins p-Rb S608 and S807, ac-E2F1, cyclin-E1, CDK2, and SSBP1. Proteins were detected in 30 tumors (T) and the paired normal tissues (N). The relative intensities of each protein in the paired dot plots were detected using ImageJ software (bottom). The analyses used a two-tailed paired Student’s t-test. b. p-Rb T821 and S780 protein levels were detected in HeLa and SiHa cells overexpressing circZFR, or circ-ZFR knockdown (sh-circ) and the corresponding controls (circ-Ctrl and sh-Ctrl, respectively). c. Immunohistochemistry of p-Rb S807 and S608 in 30 tumors (T) and the paired normal tissues (N). Left showed a representative image, and the right panel showed statistics analysis. **P< 0.01, ***P< 0.001, ****P < 0.0001. [file 13046_2021_1849_MOESM3_ESM.jpg]

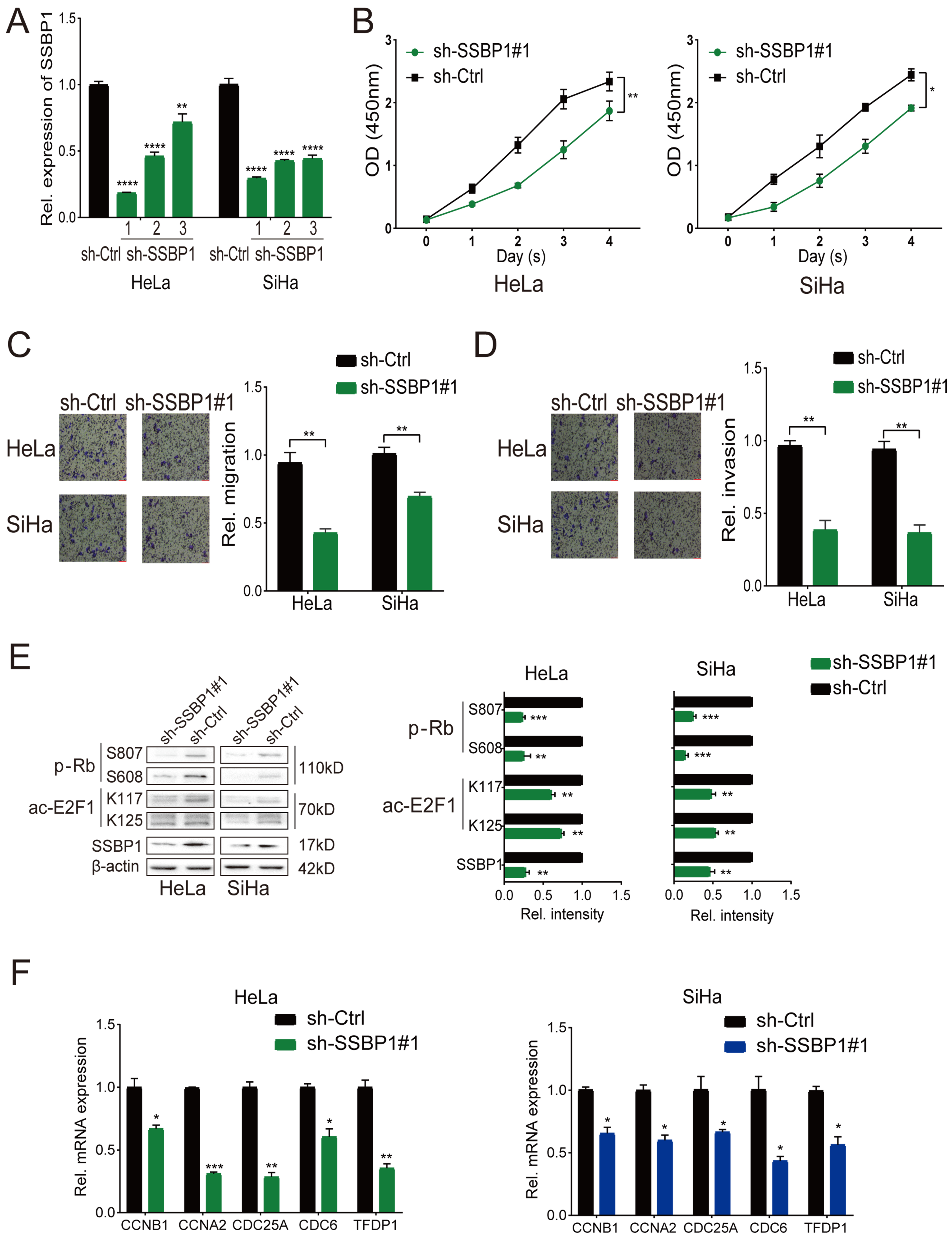

Supplement: Supplementary file 4 — Additional file 4: Fig. S4. Downregulation of SSBP1 inhibited proliferation, migration, and invasion of cervical cancer cells and inhibited Rb phosphorylation and E2F1 acetylation. a. qRT-PCR analysis of SSBP1 expression in HeLa and SiHa cells after transfection using three different shRNAs targeting SSBP1 or control-shRNA (sh-Ctrl). Sh-SSBP1#1 showed the highest knockdown efficacy. b. Downregulation of SSBP1 inhibited the proliferation ability of HeLa and SiHa cells as measured by CCK-8 assay. c-d. Knockdown of SSBP1 impeded cell migration and invasion ability of HeLa and SiHa cells as measured by transwell matrigel migration and invasion assays (Scale bar in c and d = 50 μm). e. p-Rb S608 and S807, ac-E2F1 K117 and K125, and SSBP1 protein levels were detected in HeLa and SiHa cells knocking down SSBP1 (sh-SSBP1#1) and the corresponding controls (sh-Ctrl). The relative intensities of these proteins were quantified by ImageJ (right panel). f. The mRNA expression of CCNB1, CCNA2, CDC25A, CDC6, and TFDP1 was decreased after knocking down SSBP1. *P < 0.05, **P< 0.01, ***P< 0.001, ****P< 0.0001. [file 13046_2021_1849_MOESM4_ESM.jpg]
