# Supplementary material for: The circular RNA circZFR phosphorylates Rb promoting cervical cancer progression by regulating the SSBP1/CDK2/cyclin E1 complex
Source: J Exp Clin Cancer Res. 2021 Jan 30;40:48. doi: 10.1186/s13046-021-01849-2 (PMC7846991; doi:10.1186/s13046-021-01849-2)
Supplement: Supplementary file 6 — Additional file 6: Supplementary Table 1. Primers used in qRT-PCR analysis. Supplementary Table 2. Sequence of circZFR shRNAs and negative control. Supplementary Table 3. Target sequences of SSBP1 shRNAs. Supplementary Table 4. Sequences of E2F1 siRNAs. [file 13046_2021_1849_MOESM6_ESM.docx]

**Supplementary Table 1. Primers used in qRT-PCR analysis**

| Name | Sequence (5’-3’) | |
| --- | --- | --- |
|  | Forward | Reverse |
| β-actin | CCTGGCACCCAGCACAAT | GGGCCGGACTCGTCATAC |
| CDK1 | CACAAAACTACAGGTCAAGTGG | GAGAAATTTCCCGAATTGCAGT |
| CCNE1 | TTGTGTCCTGGCTGAATGTATA | AAGGAAATTCAAGGCAGTCAAC |
| CCNB1 | GACTTTGCTTTTGTGACTGACA | CCCAGACCAAAGTTTAAAGCTC |
| CCNA2 | AGAAACAGCCAGACATCACTAA | TTCAAACTTTGAGGCTAACAGC |
| CDC25A | CGAGTCAACAGATTCAGGTTTC | CGATGAGCTGAAAGATGTCATG |
| CDC25C | GACAGGTCTCTGAACCAGTATC | TCTGGAAAGAAGTCTCTGTAGC |
| CDC6 | GTAACCTGTTCTCCTCGTGTAA | CATTCTCTTTCTTGCCTTGCTT |
| TFDP1 | CATAGACCAGAACCTTAGTCCC | GTCTCTGAGGCGTACCAATTAC |
| CDT1 | CTGTTGTACTATCATGAGCCCT | CTTGTCCAGCTTGACGTAGG |
| RRM2 | AGTGGAAGGCATTTTCTTTTCC | GCAAAATCACAGTGTAAACCCT |
| CDC45 | CCTCTTTGACTACGAGCAGTAT | TTTGAGTGATCTTGTCTTGCAC |
| TK1 | GTTCTCAGGAAAAAGCACAGAG | GTCTTTGGCATACTTGATCACC |
| RNASEH2A | ATGGTCTACGCCATCTGTTATT | CTGTATCATGTGACAGGGAGTT |
| MCM10 | CATGAATACCACTGGCATGATG | CTTTTCCTTTAGCATTCCGTCC |

**Supplementary Table 2. Sequence of circZFR shRNAs and negative control**

|  | siRNA | shRNAs | |
| --- | --- | --- | --- |
|  |  | Top strand | Bottom strand |
| sh-Ctrl | TTCTCCGAACGTGTCACGTAA | GATCCGTTCTCCGAACGTGTCACGTAATTCAAGAGATTACGTGACACGTTCGGAGAATTTTTTC | AATTGAAAAAATTCTCCGAACGTGTCACGTAATCTCTTGAATTACGTGACACGTTCGGAGAACG |
| Sh1-circ | TTTCCAAGCTGGCCCTTACGTCGTC | GATCCGTTTCCAAGCTGGCCCTTACGTCGTCTTCAAGAGAGACGACGTAAGGGCCAGCTTGGAAATTTTTTG | AATTCAAAAAATTTCCAAGCTGGCCCTTACGTCGTCTCTCTTGAAGACGACGTAAGGGCCAGCTTGGAAACG |
| Sh2-circ | CTGATTTTCCAAGCTGGCCCTTACG | GATCCGCTGATTTTCCAAGCTGGCCCTTACGTTCAAGAGACGTAAGGGCCAGCTTGGAAAATCAGTTTTTTG | AATTCAAAAAACTGATTTTCCAAGCTGGCCCTTACGTCTCTTGAACGTAAGGGCCAGCTTGGAAAATCAGCG |
| Sh3-circ | CAAGCTGGCCCTTACGTCGTCCTGA | GATCCGCAAGCTGGCCCTTACGTCGTCCTGATTCAAGAGATCAGGACGACGTAAGGGCCAGCTTGTTTTTTG | AATTCAAAAAACAAGCTGGCCCTTACGTCGTCCTGATCTCTTGAATCAGGACGACGTAAGGGCCAGCTTGCG |

sh-Ctrl: sh-control, sh1-circ: sh1-circZFR, sh2-circ: sh2-circZFR, sh3-circ: sh3-circZFR.

**Supplementary Table 3. Target sequences of SSBP1 shRNAs**

| **shRNA** | **Target sequence** |
| --- | --- |
|  |  |
| Sh-SSBP1#1 | ccAGTTTGGTTCTTGAAAGAT |
| Sh-SSBP1#2 | ctTCGTCAGTTTGTAAGACAT |
| Sh-SSBP1#3 | gcAACTAATGAGATGTGGCGA |

**Supplementary Table 4. Sequences of E2F1 siRNAs**

| **Gene** | **Sense (5’- 3’)** | **Antisense (5’- 3’)** |
| --- | --- | --- |
| Si-E2F1#1 | GUCACGCUAUGAGACCUCATT | UGAGGUCUCAUAGCGUGACTT |
| Si-E2F1#2 | GGACCUUCGUAGCAUUGCATT | UGCAAUGCUACGAAGGUCCTG |
